# CNspector: a web-based tool for visualisation and clinical diagnosis of copy number variation from next generation sequencing

**DOI:** 10.1038/s41598-019-42858-8

**Published:** 2019-04-23

**Authors:** John F. Markham, Satwica Yerneni, Georgina L. Ryland, Huei San Leong, Andrew Fellowes, Ella R. Thompson, Wasanthi De Silva, Amit Kumar, Richard Lupat, Jason Li, Jason Ellul, Stephen Fox, Michael Dickinson, Anthony T. Papenfuss, Piers Blombery

**Affiliations:** 1 Peter MacCallum Cancer Centre, 305 Grattan Street, Parkville, VIC 3000 Australia; 2 Department of Pathology, Peter MacCallum Cancer Centre, Parkville, VIC Australia; 3 Sir Peter MacCallum Department of Oncology, University of Melbourne, Melbourne, VIC Australia; 4 Department of Pathology, University of Melbourne, Melbourne, VIC Australia; 5 Department of Medical Biology, University of Melbourne, Melbourne, VIC Australia; 6 Bioinformatics Division, The Walter and Eliza Hall Institute of Medical Research, Parkville, VIC Australia; 7 Children’s Cancer Institute, University of New South Wales, Sydney, NSW Australia

**Keywords:** Cancer genomics, Molecular medicine, Cancer genomics

## Abstract

Next Generation Sequencing is now routinely used in the practice of diagnostic pathology to detect clinically relevant somatic and germline sequence variations in patient samples. However, clinical assessment of copy number variations (CNVs) and large-scale structural variations (SVs) is still challenging. While tools exist to estimate both, their results are typically presented separately in tables or static plots which can be difficult to read and are unable to show the context needed for clinical interpretation and reporting. We have addressed this problem with CNspector, a multi-scale interactive browser that shows CNVs in the context of other relevant genomic features to enable fast and effective clinical reporting. We illustrate the utility of CNspector at different genomic scales across a variety of sample types in a range of case studies. We show how CNspector can be used for diagnosis and reporting of exon-level deletions, focal gene-level amplifications, chromosome and chromosome arm level amplifications/deletions and in complex genomic rearrangements. CNspector is a web-based clinical variant browser tailored to the clinical application of next generation sequencing for CNV assessment. We have demonstrated the utility of this interactive software in typical applications across a range of tissue types and disease contexts encountered in the context of diagnostic pathology. CNspector is written in R and the source code is available for download under the GPL3 Licence from https://github.com/PapenfussLab/CNspector.

## Introduction

Detection of copy number variations (CNVs) is an important and clinically relevant part of characterizing the genomic aberrations in patients with malignancy. For example, patients with chronic lymphocytic leukaemia with a copy number loss at the *TP53* locus have inferior outcomes when treated with chemoimmunotherapy^1^ and should preferentially be treated with non-chemotherapy based approaches^2^. CNVs can be detected by a variety of laboratory methodologies including conventional cytogenetics, fluorescence *in situ* hybridisaton (FISH) and single nucleotide polymorphism (SNP) array. CNVs can also be detected from next generation sequencing (NGS) data. This is important because sequence variant detection using NGS is increasingly being utilized in the clinical diagnostic laboratory in order to improve diagnosis, refine prognosis and enhance therapeutic decision making. Therefore the analysis of copy number data generated by assays that are typically designed for the detection of sequence variants can be used to further enhance clinical decision making.

Depending on the clinical indications, NGS assays can be done either with or without enrichment that targets specific regions of interest. Despite the decreasing costs of whole genome sequencing (WGS), targeted sequencing is still the mainstay of clinical diagnostics because, unlike in discovery applications, only clinically relevant regions need be considered^3^. Enrichment is commonly performed using capture-based approaches, where DNA or RNA baits attached to magnetic beads are used to bind and pull down DNA fragments for sequencing. The total size of the captured regions varies enormously – from commercially available exome kits that enrich for all annotated coding regions^3^ to custom, cancer-specific panels that enrich for tens to hundreds of clinically important genes^4^. This paper describes a visualisation tool, CNspector, to clinically assess CNVs derived from capture-based assays of all sizes as well as from WGS approaches. In addition CNspector provides extra functionality to address the large dynamic range found in read abundance generated by targeted sequencing.

## Methods

### Architecture

**Figure 1:**
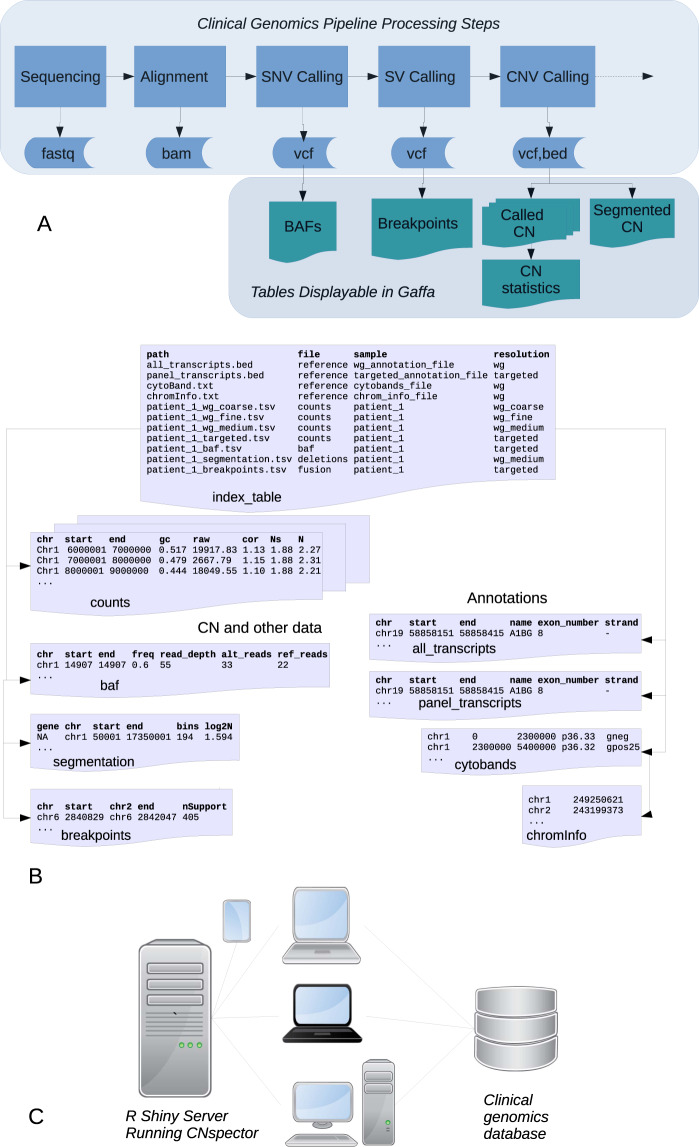
Integration of CNspector with existing clinical work flows. (**A**) Stages of processing for a clinical genomics pipeline (**B**) Pre-built tables at all viewable scales allow rapid rendering in CNspector (**C**) CNspector is implemented with a client-server architecture allowing it to be used on any device with a web browser. Integration with an existing clinical genomics system can be done by linking to a URL identifying the sample and loci to be viewed.

The inputs required by CNspector are available in most modern clinical genomics pipelines where sequence variants, CNV and SV calls are routinely generated for all samples. This is normally followed by loading into a database for access by clinical curation and reporting applications as shown in Fig. 1(A). CNspector reads in tracks as tab-separated text tables which can come either from actual files, from URLs or other R text-mode connections^5^ making them easy to generate from a database query if required. Each instance of CNspector needs one initial index table whose rows specify all the other tables that may be displayed plus associated meta-data. Figure 1(B) shows a typical index table for one sample – the first two entries are the clinical transcript annotations for all regions and for targeted areas respectively. The next two entries are cytoband co-ordinates and chromosome details required for display. There are four rows specifying tables containing copy number (CN) calls binned at four different resolutions and the final two rows specify tables with segmented CN calls and SV breakpoints. CNspector is not tied to any particular pipeline or variant caller. It only needs an index table pointing to valid annotations and sample tables containing a minimal set of columns (shown in Fig. 1(B)).

We have implemented CNspector as a client-server architecture using R-Shiny^6^ (Fig. 1(C)), allowing it to be used from any device with a web browser without installation or maintenance of special software on user machines. Alternatively, for users comfortable using R Studio, the server can be run locally on the same machine as the browser. Using a Shiny running on a server rather than a browser-based implementation in java script avoids browser portability/optimisation issues and enables use of R’s extensive library of numerical and bioinformatics packages. We have minimised computation on the server-side and a server with a Xeon E5-2660 and 48 GB RAM is able to service the needs of 20–30 staff members working in a diagnostic pathology department with throughput of 100–200 samples per week. Pathologists and scientists examining patient data produced from the Peter MacCallum Cancer Centre (PMCC) clinical genomics pipeline use a curation and reporting system, PathOS^7^ to curate and annotate the relevant called variants. From here they can click on a link that quickly displays the patient data in CNspector for further analysis. Of note, CNspector is not tied to any particular variant curation platform or bioinformatics pipeline. The light-weight integration to PathOS could be trivially replicated in analogous systems.

### Workflow

**Figure 2:**
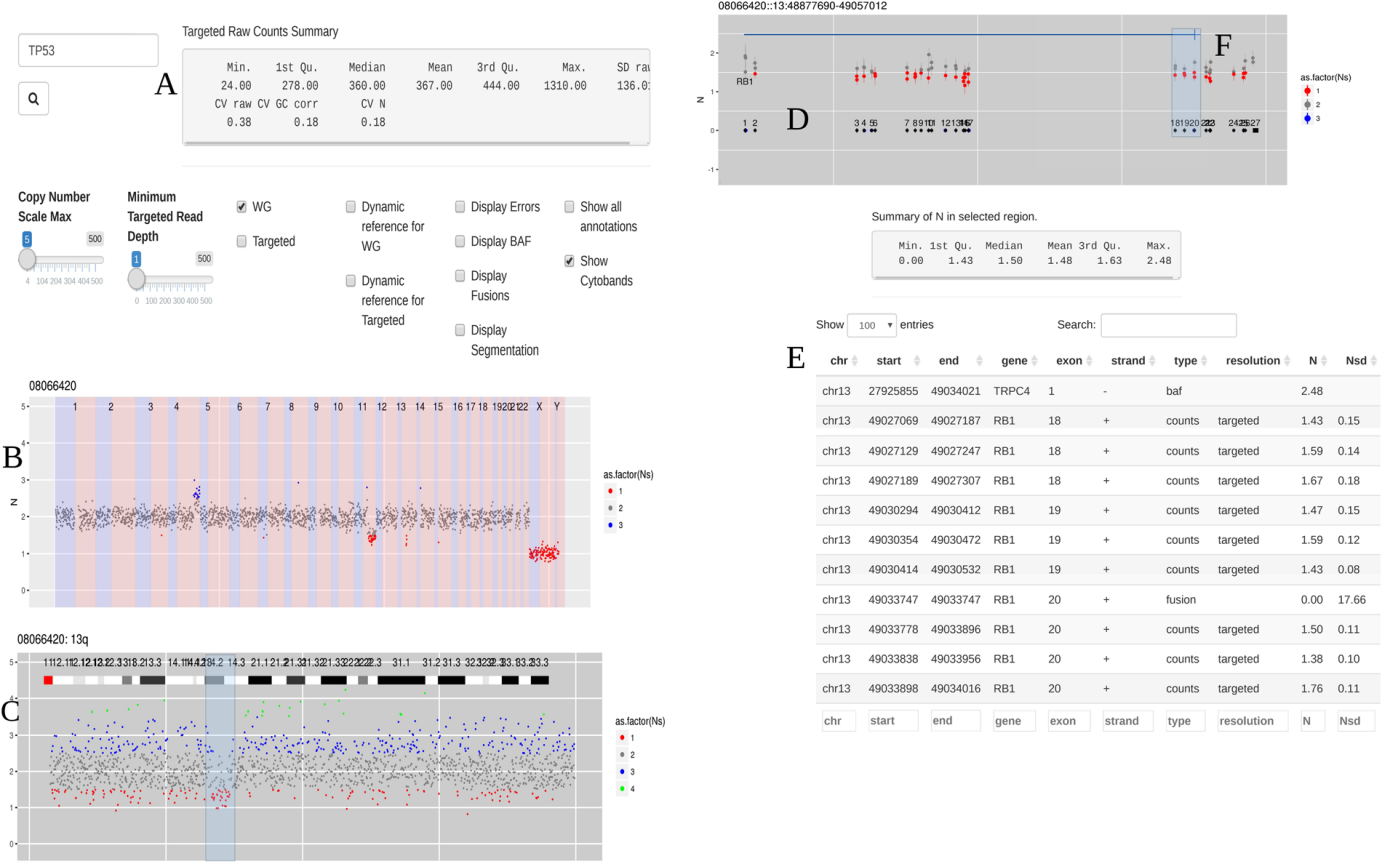
Steps when using CNspector to assess CNVs (**A**) An initial QC step consists of checking coverage and sample noise for raw read counts and estimated copy number (**B**) CN changes at the chromosome and chromosome arm level are checked in the top plot, made of 1MB bins. (**C**) Smaller scale gains and losses such as the one on 13q can be checked on the second plot. (**D**) Selecting regions of interest in the second plot enables assessment of CN with respect to exon-level annotations in the third plot. (**E**) Selecting points of interest in this plot displays relevant details such as copy number estimate at each bait, error estimate based on reference samples and estimated read abundance. (**F**) Supporting evidence may also be viewed – in this example one end of a breakpoint near exon 20 of RB1 is shown. Other sorts of supporting evidence would be boundaries of segmented regions and B-allele frequencies.

The information required to perform the tasks required for clinical assessment of CNVs is presented logically starting from the top of the user interface. The first summary statistics box (Fig. (2A)) is dynamically generated when a sample is plotted and for target areas it shows the variation in the uncorrected read abundance followed by GC corrected version, and finally, the called copy number (CN). The next step is to read off chromosome arm-level CN changes from the 1 Mb bins displayed in the first plot. This is most easily done with the targeted track removed by unchecking the corresponding check box (SI Video 1). If there is sample contamination or heterogeneity, this is typically apparent here. Red triangles at the top of the plot indicates overflow and so the plot scaling should be adjusted until these disappear to reveal focal amplifications. At this point turning on the targeted track will display bait-level CN in all three panels.

CN changes from the top panel can be further investigated by clicking on the containing chromosome arm. This causes the selection to be rendered in the middle panel using 50 kb bins (Fig. 2(C)). Highlighting an area of interest in this panel causes it to be rendered in the bottom panel with 5 kb bins. Depending on the area this will often display more than one gene so an option exists to double click near any gene to zoom in on it, at which points its exons will fill the bottom plot (Fig. 2(D)). Double clicking again returns to the originally selected area. Finally, selecting features in the bottom plot causes the raw data for those features to be placed into a table and summary of the CN (or relevant quantity stored in the column N) is shown (Fig. 2(E)). This is helpful when following up QC issues or checking supporting evidence from breakpoint calls and B-allele frequencies (Fig. 2(F)).

### Interactive visualization at multiple scales with contextual data

Regardless of NGS assay type described above, read abundance is modulated by a variety of technical artefacts from different parts of the workflow. Examples are PCR bias arising from the DNA fragment’s length or GC content^8^ and factors affecting alignment such as the reference genome’s mappability and fidelity^9^ and deviation of the sample from the reference genome due to disease or just normal population-wide variability^10^. For assays involving enrichment, the coverage in targeted regions is additionally dependent on other factors such as pull down efficiency, non-uniform bait distributions, competitive binding by homologous sequence and flanking-region effects^11^. CNV callers correct for much of this variation but false positives remain a problem^12^. Consequently, in clinical reporting there is a requirement to visualise CNVs with reference to contextual data to provide supporting evidence and interpret biological implications. Note that this contextual data comes not only from the clinical variant calling pipeline for the same sample (for example SNVs, indels, breakpoints), but also from annotated historical samples that have been reported previously.

To address the issues of contextualisation and interpretability we bin the CN calls at different scales required to capture the relevant biology and display them simultaneously at different levels of magnification. We implement this in a multi-scale browser where CN estimates from four different levels of detail can be displayed simultaneously in three different plots zoomed to match the levels of detail. By preparing all displayable tables in advance and using dynamically-generated browser-based plots, it is possible to quickly navigate between the different scales by clicking on the relevant chromosome arm, selecting a desired genomic region or double clicking near a gene of interest. The bins sizes accommodate the full dynamic range from all assays – starting at 1 Mb when displaying genome-wide data and going all the way down to bait-size when looking at the targeted exon level – so that users can view the most accurate CN calls and the most precise breakpoint estimates simultaneously.

### Multi-sample Mode

A multi-sample mode is available that enables samples to be compared with each other or with other groups of samples. This is turned on automatically when more than one sample is included in the index table and enables the option to estimate CN using a reference that is dynamically generated from samples selected in the index file. The selection is performed interactively by the user at run time, making it simple to call CN against references chosen on the basis of matching technical artefacts. This capability is especially useful during assay-development when references samples may not be available nor the effects of technical variability known in advance (SI Fig. 1).

Median and median absolute deviation are used to produce robust estimates for the reference mean and reference standard deviation (SD) respectively, as described in SI Section S1. The use of these robust estimators enables the inclusion of tumour samples into the reference set without introducing bias.The SD estimates can optionally be plotted as error bars, although it should be noted that they underestimate the SD of the CN since they only represent the contribution from the sample being tested and not from the reference set that was used to estimate them. Even so, with enough reference samples they become a good lower bound and useful both for assessing significance and finding assay trouble spots.

## Results

### Application to patient samples

In order to demonstrate the utility of CNspector across a range of sample types in clinical practice we present patient samples sequenced from both formalin-fixed paraffin embedded (FFPE) and fresh frozen tissue, from somatic and germline samples and with and without target enrichment. For all patient samples presented, relevant DNA library characteristics are summarized in SI Table 1, bait-based enrichment where applicable, is summarized in SI Table 2 and bioinformatics details for generation of CN and other genomic features are described in SI Section S1. Bioinformatic details for CNspector internals are described in SI Section S2. A brief walk-through of each patient sample in CNspector can be found in SI Video 1.

### Focal deletions in germline DNA from blood

**Figure 3:**
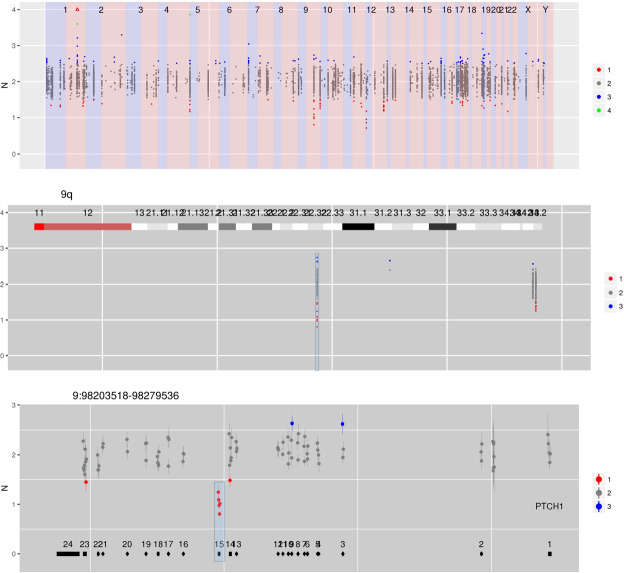
CN estimates for enriched regions – in this case genes in which germline mutations confer an increased risk of cancer – show a focal loss at exon 15 in PTCH1.

Figure 3 is a germline sample derived from peripheral blood containing a focal heterozygous deletion of exon 15 of the tumour suppressor gene *PTCH1*. Germline alterations in *PTCH1* underlie the nevoid basal cell carcinoma syndrome or Gorlin syndrome, which is an autosomal dominant disorder predisposing to basal cell carcinomas. Large genomic rearrangements in *PTCH1* occur in ~6–21% of individuals diagnosed with nevoid basal cell carcinoma syndrome^13–15^.

### Chromosome and exon level CNVs in tumour DNA from bone marrow

**Figure 4:**
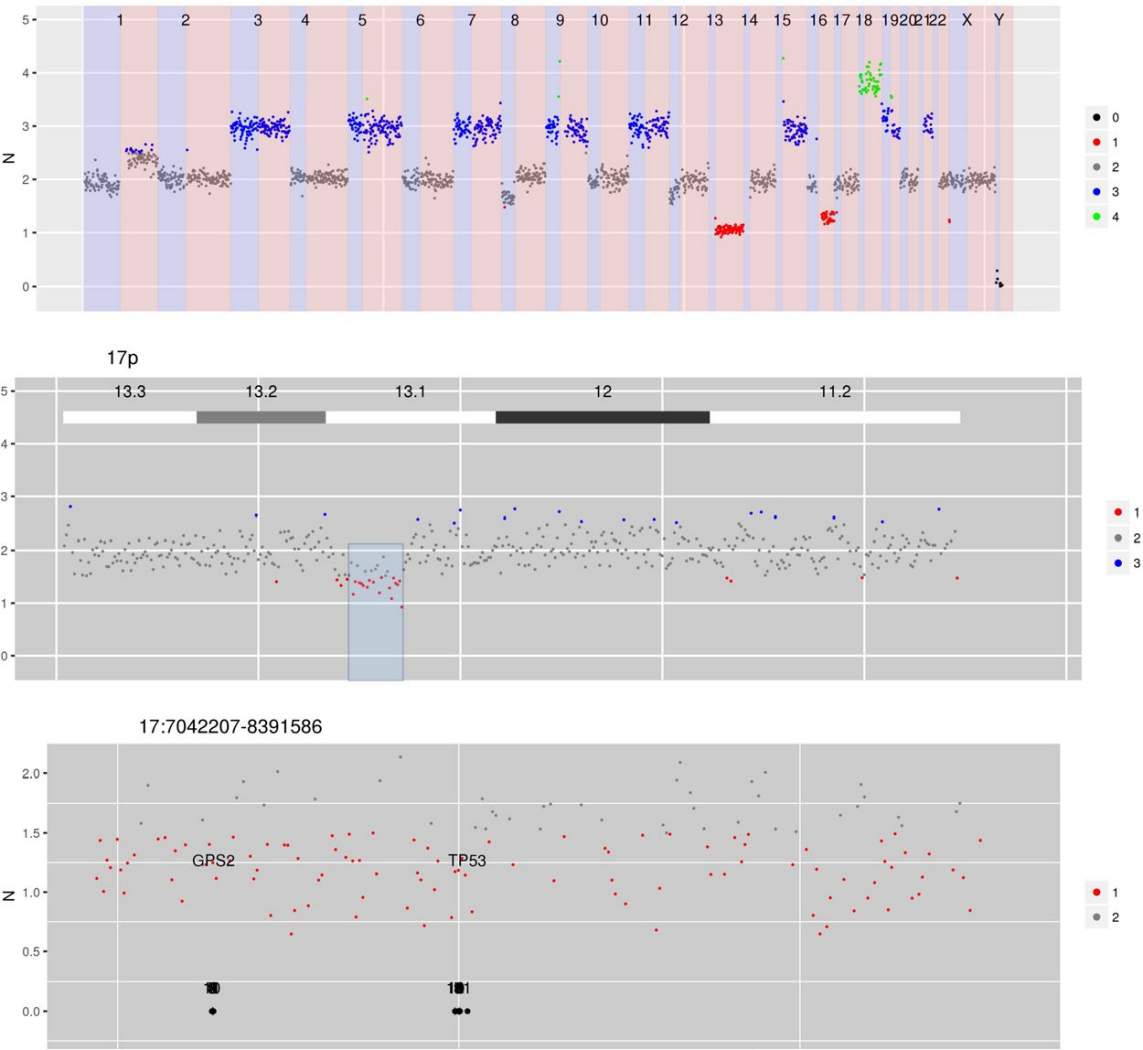
CN estimates for WG derived from targeted sequencing of a myeloma patient. Common chromosome duplications can be seen on odd chromosomes along with sub-clonal CNV on 1q, 8p and 16q. A focal deletion involving TP53 is hi-lighted for display in the bottom panel.

Figure 4 shows copy number data was obtained from a patient with multiple myeloma from DNA extracted from a bone marrow aspirate. The copy number data demonstrates the typical non-random chromosomal gains that are associated with hyperdiploid multiple myeloma (involving chromosomes 3, 5, 7, 9, 11, 15, 19 and 21). In addition a monosomy 13 is demonstrated which is, again, a typical aneuploidy associated with multiple myeloma. In addition to these large scale genomic events, a highly focal deletion on chromosome 17p can be seen involving the *TP53* locus. *TP53* copy number changes are associated with inferior outcomes in patients with myeloma^16^. This case demonstrates the ability of CNspector to interrogate chromosomal level aneuploidies and focal, sub-chromosomal loss of tumour suppressor genes efficiently and intuitively.

### CNVs and loss of heterozygosity in circulating tumour DNA from blood

**Figure 5:**
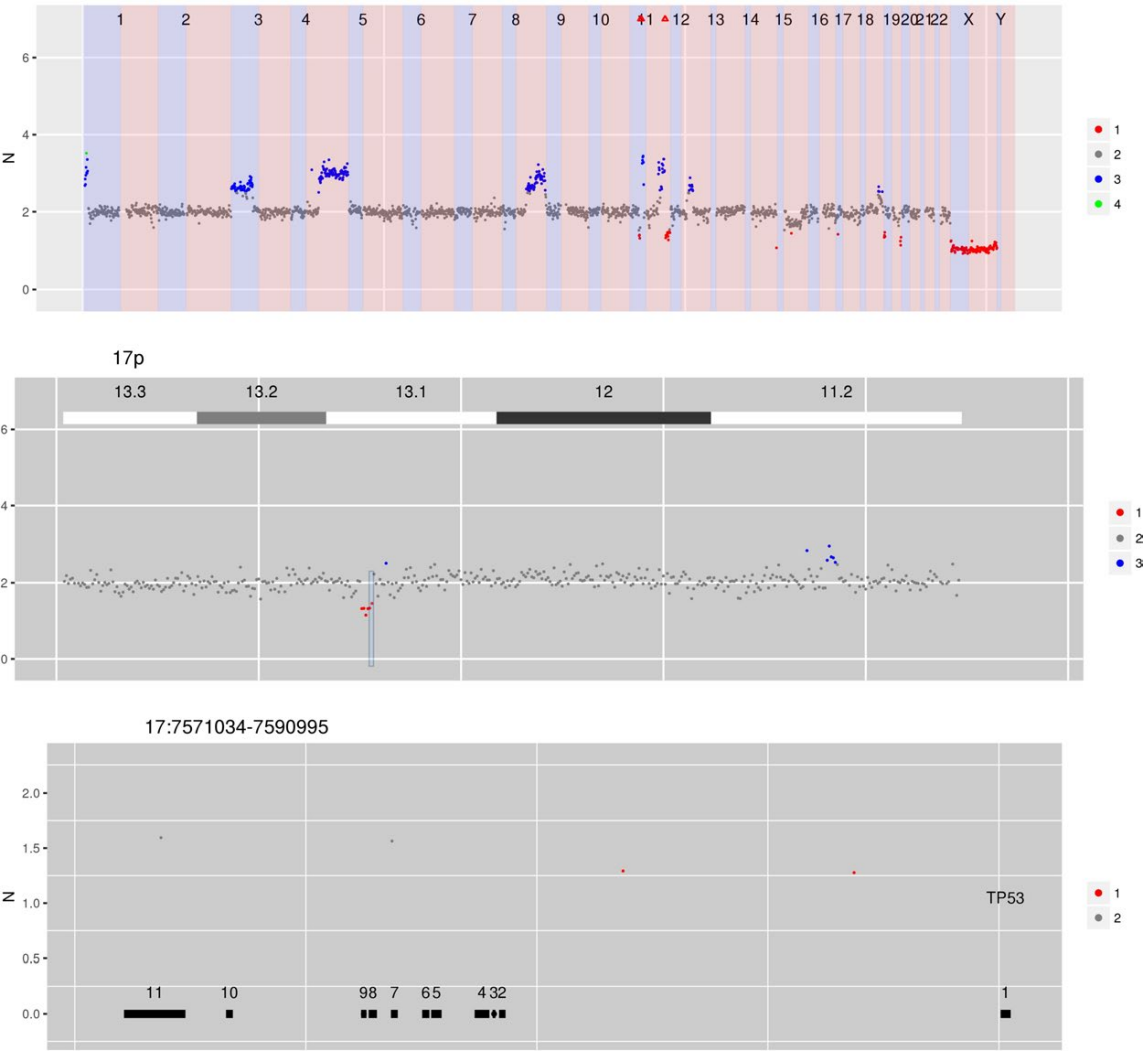
CN estimates for WG derived from targeted sequencing of circulating cell-free DNA from a patient with diffuse large B cell lymphoma. Several focal amplifications and deletions are apparent, including the hi-lighted focal deletion spanning TP53.

Figure 5 shows copy number data obtained from a patient with diffuse large B-cell lymphoma from circulating tumour DNA extracted from the patient’s plasma. As can be easily appreciated from the copy number data visualisation, two high-level amplifications are present. Confirmation of overexpression of genes involved in these amplification was performed by gene expression profiling on a tumour specimen. In addition, a highly focal copy number change involving *TP53* is seen and CN-neutral loss of heterozygosity involving chromosome 1p and 2 (Fig. 5).

### Complex CNVs and problematic samples

Interpretation of copy number data from FFPE-derived DNA can be particularly challenging due to the noise introduced by poor quality starting DNA^17,18^. SI Figure 6 is an FFPE sample from a high grade ovarian cancer with the copy number scale adjusted to highlight a focal amplification of *KRAS* at 10–30 copies. At default scale settings it is also possible to detect CN gains on significant portions of most chromosomes and losses on parts of chromosomes 4q, 5q, 8p, 10q, 16q and 17q.

In SI Figure 7, CNspector is used to analyse a myeloma sample WG-sequenced to a coverage depth of just over 2×. Despite the low coverage, it is still possible to zoom in and assess small features such as the focal amplification highlighted on the chemokine genes on chromosome 17q.

SI Figure 8 demonstrates how assessing complex genomic arrangements benefits from display at multiple scales. Chromosome 12 has undergone a series of genomic events that have left a patchwork of segments with copy numbers at 1, 3, 5 and beyond. Within this busy context there are small segments of much higher CN encompassing clinically significant genes. CNspector makes it simple to assess these small focal amplifications, as is shown in the case of *CDK4*.

In the case of whole exome sequencing the density of targeted areas means that B-allele frequencies can be used by model-based methods to estimate allele-specific CN and tumour purity^19,20^. SI Figure 9 demonstrates how CNspector can also be used to read off similar information in the case of an Agilent V5 exome. For simple situations such as the one shown, this enables a quick visual confirmation to be made. Alternatively, when model-based methods are unable to resolve ambiguity and are confounded, CNspector may be used to investigate the source of the problem.

### Novel applications enabled by multi-sample mode

Multi-sample mode is particularly useful for comparing two samples directly to check for small differences. In this case one of the samples is chosen as the reference and the ratio of the CN between the two is displayed. Applications for this capability include comparing samples from two time points to follow patient progression (SI Fig. 2), comparing negative controls from different sample batches to examine technical artefacts (SI Fig. 3) and troubleshooting suspected contamination (SI Fig. 4). Multi-sample mode can, in fact, be used to view ratios of any counts-based data provided that it can be scaled appropriately. For example it is possible to view differential expression between RNA-seq samples, allowing an exploration of the impact of chromosome-wide CN changes on transcript abundance (SI Fig. 5).

### Integration of existing copy number callers to work with CNspector

The expected use case for CNspector is to view CNV data produced by existing clinical bioinformatics pipelines. To this end we provide scripts that generate and then convert the outputs from existing CN callers into tables suitable for display by CNspector. While there are many published CN callers in use, only a small proportion of them are able to produce WG CN calls at all scales using both small targeted panels and WGS. We have chosen three representative callers that meet these criteria^11,21,22^ and provide R scripts that can input output from these and hence provide a template for doing the same for other callers. CNspector requires CN calls at four different resolutions. When a caller can accept genomic regions (bins) specified by the user^22^ then the corresponding CN calls are generated by re-running the caller for each bin size. When the caller is unable to accept user-defined bins^21,22^ then the conversion script needs to re-bin the CN calls to at each displayed scale.

Using these scripts to generate and import data tables from the chosen callers enables us compare the performance of CNspector to the visualization methods that would otherwise be used. SI Figure 10 shows typical screen shots of data visualised with CNspector and with the default option typically used for the callers that we ran. SI Table 3 breaks this down in terms of functionality. As expected there is substantial improvement over other options. Perhaps less expected is that CNspector is also able to improve the CN calls themselves by using multi-sample mode to improve normalisation (SI Figure 11).

## Discussion

Uncontrolled variation in read abundance means that false positives continue to be an issue for calling CNVs^17,18^. Consequently, for clinical applications, putative CNVs normally require verification – usually by (i) plotting them alongside contextual data to provide supporting evidence and (ii) applying filtering using prior knowledge to improve interpretability. Examples of contextual data and filtering include:

- B-allele frequencies to check for sample purity and heterogeneity, to rule out homozygosity or, in the case of severe aneuploidy, to confirm that the diploid state has been correctly assigned.
- Segmented CN to help identify small apparent-changes in CN in the presence of heterogeneity.
- Break point calls supported by read-pairs or split reads to give support to those predicted by segmented read abundance
- Error bars to allow comparison with assay-wide variation and estimation of statistical significance.
- Per-assay blacklists that can be used to selectively mask recurrent false positives
- Per-disease whitelists than can be used to selectively display only clinically relevant lesions for further consideration.
- Annotations that have been filtered to show only clinically relevant transcripts. This, in addition to optionally limiting display to annotations for those genes being reported, removes unnecessary clutter when assessing patient samples.

Uniquely, CNspector can use all of this extra information if it is available (SI Table 3). The extra information normally is available in a clinical workflow due to the high sample throughput and the extra time available for sample curation (in contrast to discovery or research workflows); for example in a clinical setting there are usually historical samples that can be used for background read depth and variance estimation as well as a supply of previously-curated and or orthogonally-tested samples that can be used to generate per-assay black lists.

CNspector’s ability to display and navigate at three different levels makes it possible to assess a patient sample at the best possible CN resolution for chromosome-scale variation while simultaneously viewing at the best possible genomic resolution for exon-scale variation, thereby making the most of the high dynamic range in coverage present in targeted sequencing assays.

Finally, the flexibility of CNspector to interactively display and highlight differences between selected samples and or groups of samples makes it ideal for use in a range of tasks required for assay development and debugging. For example, in our case this has made it possible to use CNspector, without modification, to interpret results from circulating tumour DNA, find contamination from circulating tumour cells, observe incremental CNVs following patient treatment and assess loci-dependent batch effects over time.

## Conclusions

The application of NGS assays to clinical genomics is driving rapid expansion in the areas of clinical testing and personalised medicine. As the price of sequencing drops, the catalog of known causal variants increases and more targeted therapies become available for treatment, this process is likely to accelerate. Interpreting the genetic lesions in a patient DNA sample and assessing their clinical significance remains one of the more challenging and expansive steps in the process. In the case of sequence variants, their well-characterised error profile, the more mature algorithms used to call and interpret their effect and the consequently greater literature have significantly improved and automated analysis^23^. The literature and analysis for SV’s and CNVs on the other hand are far less mature. Moreover, the processes generating false positives arising from short read data, especially when using targeted enrichment makes development of algorithms for automatic analysis challenging. As a result the clinical curation of CNVs and SVs requires plotting the putative lesion in the context of all available relevant evidence in order to interpret the nature and clinical significance. We have developed a multi-scale browser specifically for this task that accelerates and improves interpretation by conveniently presenting the necessary genomic features required for clinical reporting. Further, we have demonstrated that the combination of flexibility and features not found together elsewhere make CNspector uniquely useful for interpreting CNV in a range of commonly-encountered and challenging clinical situations.

## Availability and requirements

Project name: CNspector.

Project home page: https://github.com/PapenfussLab/CNspector.

Operating system: Platform independent.

Other requirements: R >= 3.4.0, R::shiny >= 1.0.5.

Licence: GNU GPL3.

## Supplementary information

Supplementary Information

SI Video 1

## Data Availability

CNspector is written entirely in R and the source code is available for download under the GPL3 Licence from https://github.com/PapenfussLab/CNspector. A server running CNspector loaded with the figures from this paper can be accessed at https://shiny.wehi.edu.au/jmarkham/CNspector/index.html. The patient data displayed in the figures is not publicly available due to the presence of potentially genotyping information, but de-identified versions are available from the corresponding author on reasonable request.
