# Publisher Correction: CNspector: a web-based tool for visualisation and clinical diagnosis of copy number variation from next generation sequencing

**DOI:** 10.1038/s41598-020-67205-0

**Published:** 2020-06-23

**Authors:** John F. Markham, Satwica Yerneni, Georgina L. Ryland, Huei San Leong, Andrew Fellowes, Ella R. Thompson, Wasanthi De Silva, Amit Kumar, Richard Lupat, Jason Li, Jason Ellul, Stephen Fox, Michael Dickinson, Anthony T. Papenfuss, Piers Blombery

**Affiliations:** 1 Peter MacCallum Cancer Centre, 305 Grattan Street, Parkville, VIC 3000 Australia; 2 Department of Pathology, Peter MacCallum Cancer Centre, Parkville, VIC Australia; 3 Sir Peter MacCallum Department of Oncology, University of Melbourne, Melbourne, VIC Australia; 4 Department of Pathology, University of Melbourne, Melbourne, VIC Australia; 5 Department of Medical Biology, University of Melbourne, Melbourne, VIC Australia; 6 Bioinformatics Division, The Walter and Eliza Hall Institute of Medical Research, Parkville, VIC Australia; 7 Children’s cancer institute, University of New South Wales, Sydney, NSW Australia

Correction to: *Scientific Reports* 10.1038/s41598-019-42858-8, published online 23 April 2019

The original version of this Article contained errors in the Abstract.

“CNspector is written in R and the source code is available for download under the GPL3 Licence from https://github.com/PapenfussLab/CNspector. A server running CNspector loaded with the figures from this paper can be accessed at https://shiny.wehi.edu.au/jmarkham/CNspector/index.html.”

now reads:

“CNspector is written in R and the source code is available for download under the GPL3 Licence from https://github.com/PapenfussLab/CNspector.”

Additionally, the data availability statement contained errors.

“CNspector is written entirely in R and the source code is available for download under the GPL3 Licence from https://github.com/PapenfussLab/CNspector. A server running CNspector loaded with the figures from this paper can be accessed at http://115.146.86.255/CNspector/index.html. The patient data displayed in the figures is not publicly available due to the presence of potentially genotyping information, but de-identified versions are available from the corresponding author on reasonable request.”

now reads:

“CNspector is written entirely in R and the source code is available for download under the GPL3 Licence from https://github.com/PapenfussLab/CNspector. A server running CNspector loaded with the figures from this paper can be accessed at https://shiny.wehi.edu.au/jmarkham/CNspector/index.html. The patient data displayed in the figures is not publicly available due to the presence of potentially genotyping information, but de-identified versions are available from the corresponding author on reasonable request.”

These errors have now been corrected in the PDF and HTML versions of the Article.

