# Supplementary material for: CNspector: a web-based tool for visualisation and clinical diagnosis of copy number variation from next generation sequencing
Source: Sci Rep. 2019 Apr 23;9:6426. doi: 10.1038/s41598-019-42858-8 (PMC6478945; doi:10.1038/s41598-019-42858-8)
Supplement: Supplementary file 1 — Supplementary Information [file 41598_2019_42858_MOESM1_ESM.pdf]

Supplementary Information

# CNspector: a web-based tool for visualisation and clinical diagnosis of copy number variation from next generation sequencing

John F. Markham[1,2,3,6], Satwica Yernini[2,3], Georgina L. Ryland[2,3], Huei San Leong[2,3], Andrew Fellowes[2], Ella R. Thompson[2,3], Wasanthi De Silva[2,3], Amit Kumar[1,6,7], Richard Lupat[1], Jason Li[1], Jason Ellul[1], Stephen Fox[2,3,4], Michael Dickinson[2,3,4], Anthony T Papenfuss[1,3,5,6]* and Piers Blombery[2,3,4]*

[1] Peter MacCallum Cancer Centre, 305 Grattan Street, Parkville, VIC 3000, Australia.

[2] Department of Pathology, Peter MacCallum Cancer Centre, Parkville, VIC, Australia.

[3] Sir Peter MacCallum Department of Oncology, University of Melbourne, Melbourne, VIC, Australia.

[4] Department of Pathology, University of Melbourne, Melbourne, VIC, Australia.

[5] Department of Medical Biology, University of Melbourne, Melbourne, VIC, Australia.

[6] Bioinformatics Division, The Walter and Eliza Hall Institute of Medical Research, Parkville, VIC, Australia.

[7] Children's Cancer Institute, University of New South Wales, Sydney NSW, Australia.

# Supplementary Figures

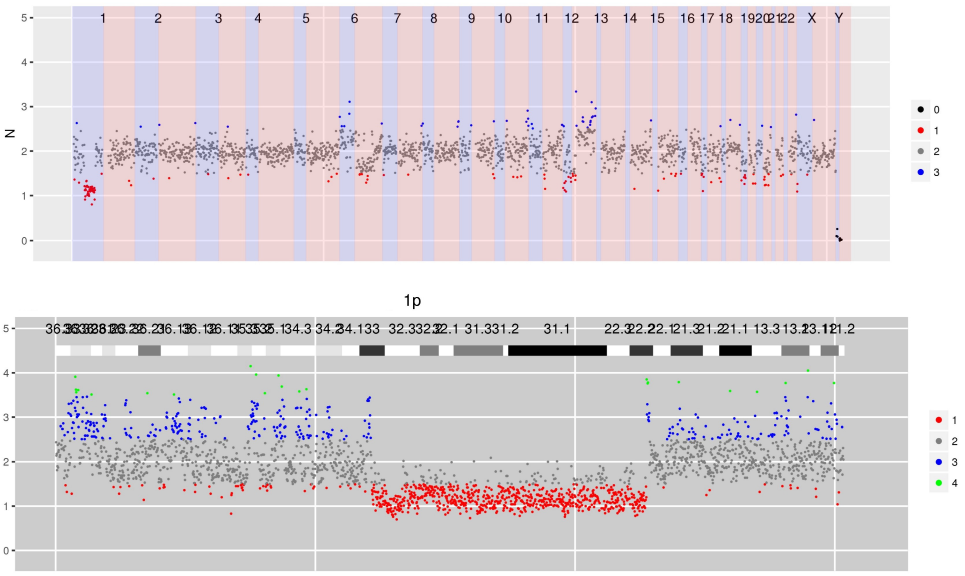


(a)

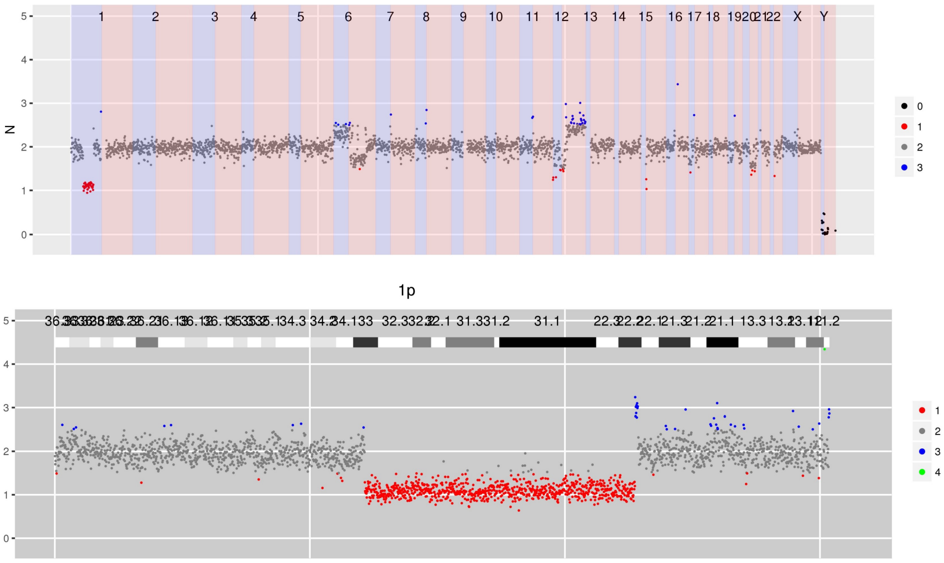


(b)

*Figure 1 (a) CN estimates for WG derived from targeted sequencing of circulating tumour DNA (ctDNA) using a reference made from a batch of normal cellular DNA samples. (b) CN estimates for the same sample using multi-sample mode to construct reference made from other ctDNA samples. The resulting CN estimates are less noisy because the technical artefacts in other ctDNA samples are a better match than the batch of normal samples.*

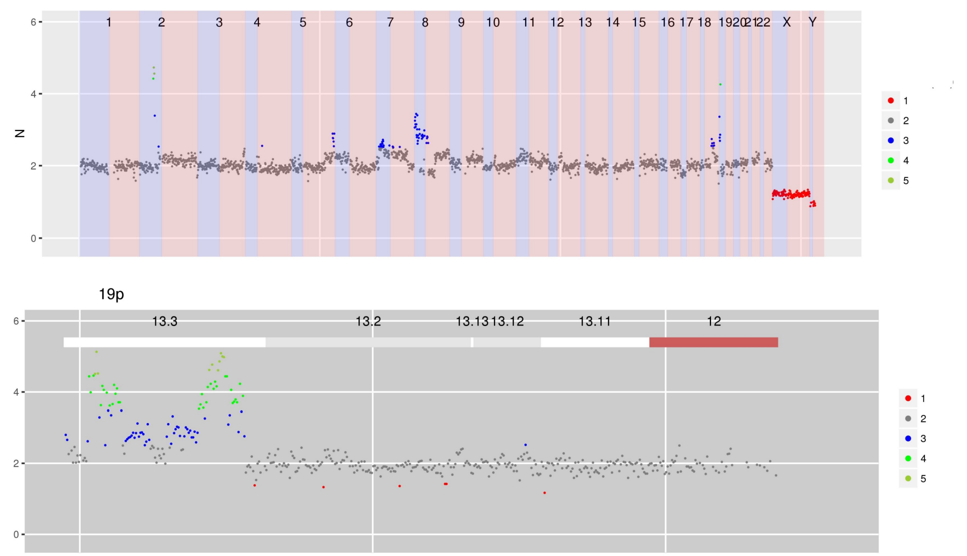


(a)

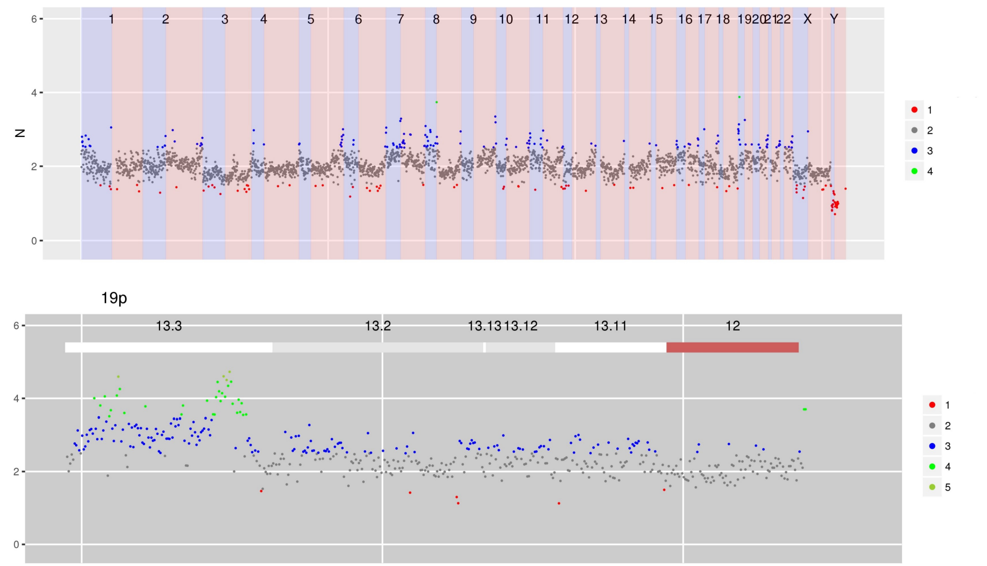


(b)

*Figure 2 (a) CN estimates for WG derived from targeted sequencing of ctDNA containing focal amplifications at chromosomes 2p, 8p and 19p. (b) Using multi-sample mode to display this sample scaled by a previous sample obtained from an FFPE block shows that the amplifications in chromosomes 2p and 8p are shared but that amplification in chromosome 19p is only in the second sample.*

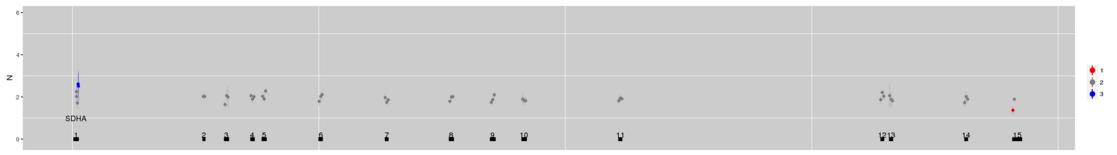


(a)

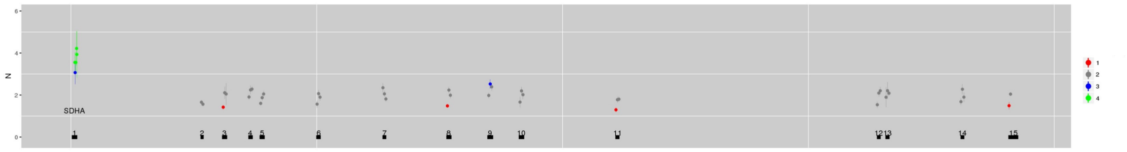


(b)

*Figure 3 Using multi-sample mode it is possible to use negative control samples from different sequencing batches to explore evolution of technical artefacts. (a) The CN estimate for the gene SDHA for NA12878 derived using references from germline samples of a similar vintage. (b) Estimates derived instead from the same sample sequenced 14 months later.*

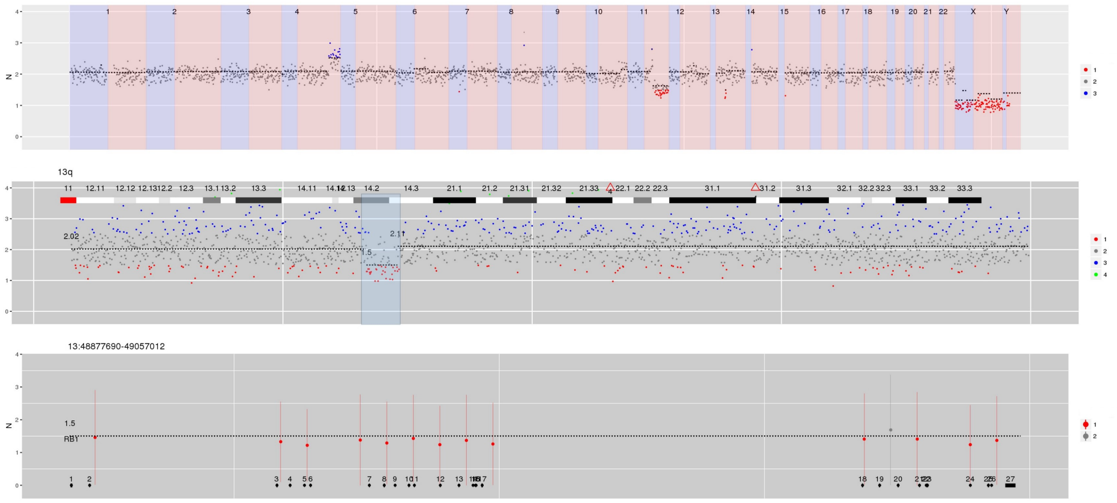


*Figure 4 CN estimates for WG derived from targeted sequencing of a germline sample from a patient being screened for mutations that confer an increased risk of cancer. Inspection of the sample shows contamination which is consistent with chronic lymphocytic leukaemia - for example the highlighted deletion of RB1 as well as the deletion in 11q containing ATM.*

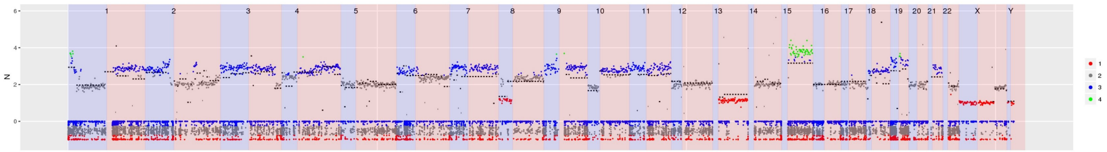

*Figure 5  CN estimates for WG derived from targeted sequencing is plotted using an auxilliary track in conjunction with BAFs and segmented differential expression obtained from RNA-seq. Averaging over differential transcript abundance across segments shows concordance with CN derived from DNA-seq while BAFs derived from RNA-seq give indications of homzygosity and clonality.*

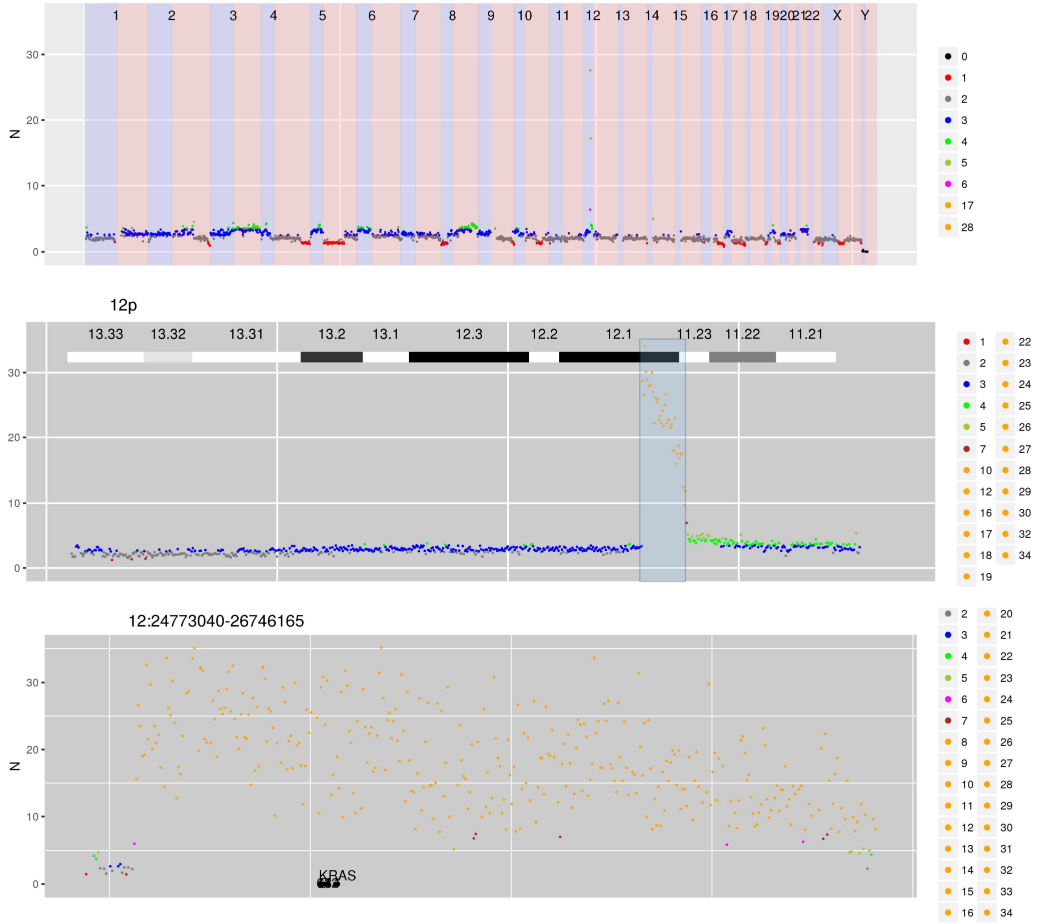


*Figure 6 CN estimates for WG derived from targeted sequencing of high grade ovarian cancer. The scale has been adjusted to enable visualisation of a focal amplification of KRAS to 10-30 copies.*

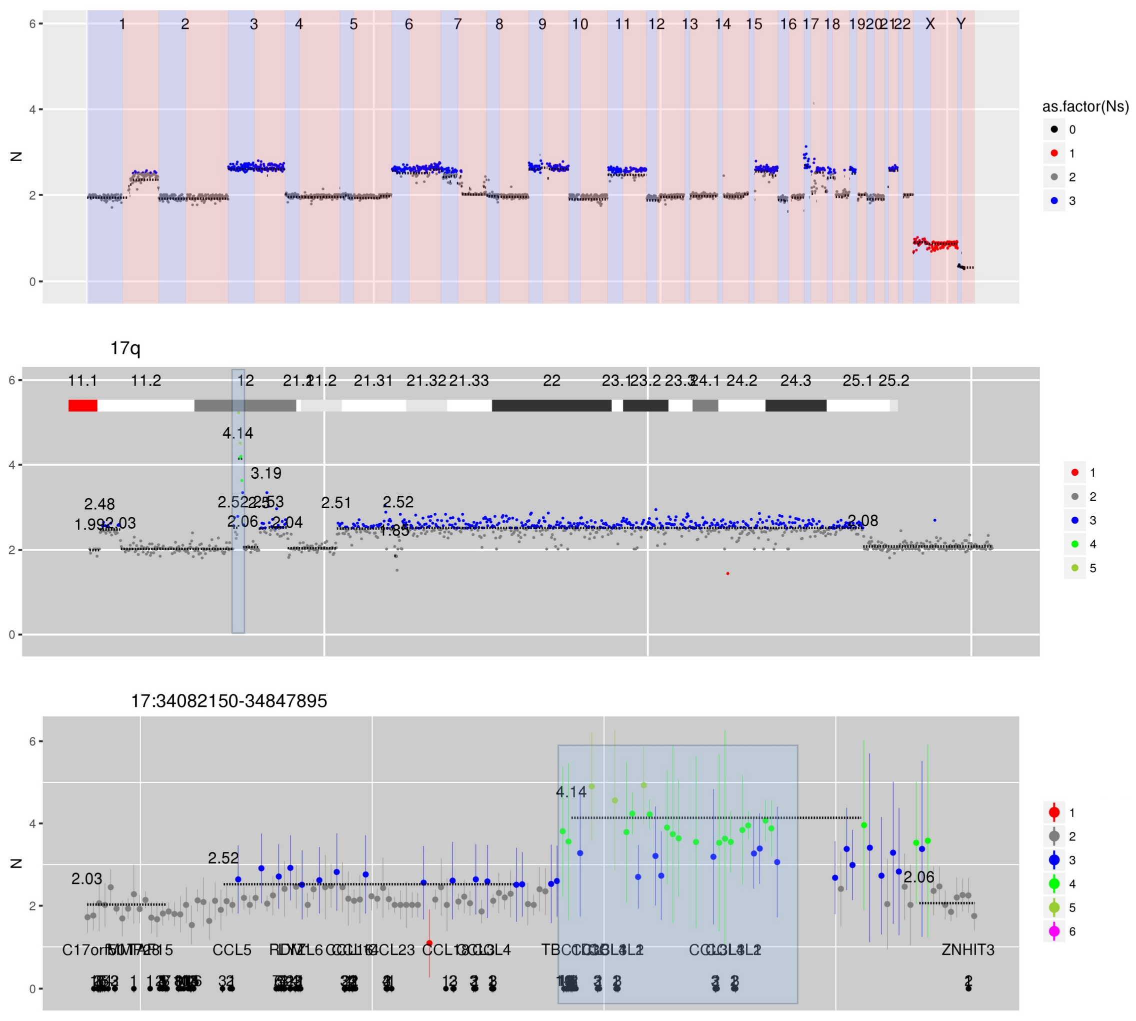


Summary of N in selected region.

| Min. | 1st Qu. | Median | Mean | 3rd Qu. | Max. |
|---|---|---|---|---|---|
| 2.60 | 3.46 | 3.81 | 3.85 | 4.23 | 5.36 |

Figure 7 CN estimates for WG derived from LC-WGS sequencing of a myeloma sample. A focal amplification of a range of chemokine genes is highlighted on chromosome 17q.

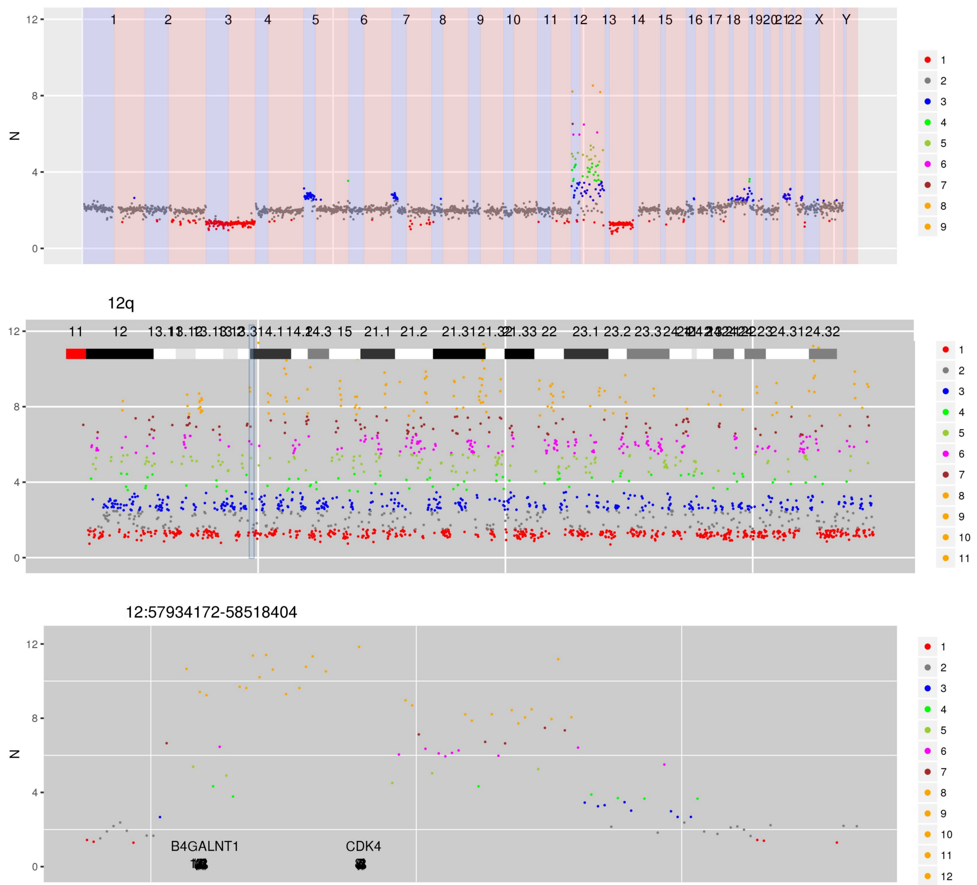


*Figure 8 CN estimates for WG derived from targeted sequencing of a likely de-differentiated liposarcoma. A complex genomic re-arrangement on chromosome 12 has caused segments of amplification at CN 3, 5 and beyond. Highly amplified regions on chromosome 12 include 12p13.31-13.2 (CCND2, ~14 copies), 12q14.1 (CDK4, ~13 copies), 12q15 (MDM2, ~10 copies), 12q24.22 (TBX3, ~11 copies).*

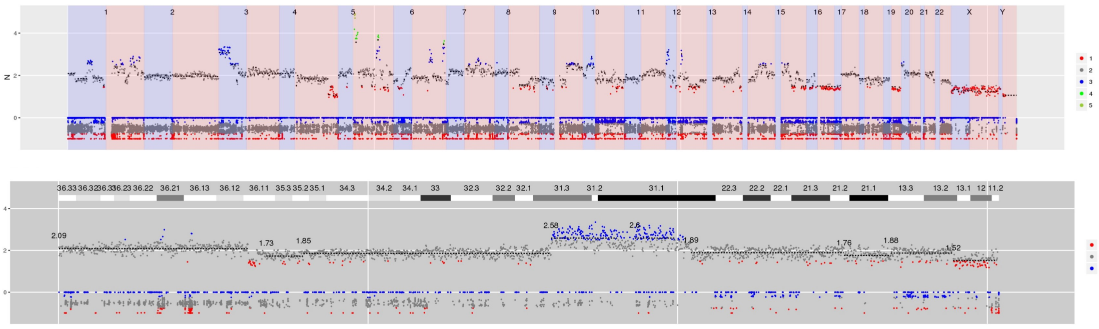

*Figure 9. CN estimates for WG derived from targeted sequencing using the Agilent V5 exome kit. Copy number neutral loss off heterozygosity is apparent in parts of 1p, 10q and 12q.*

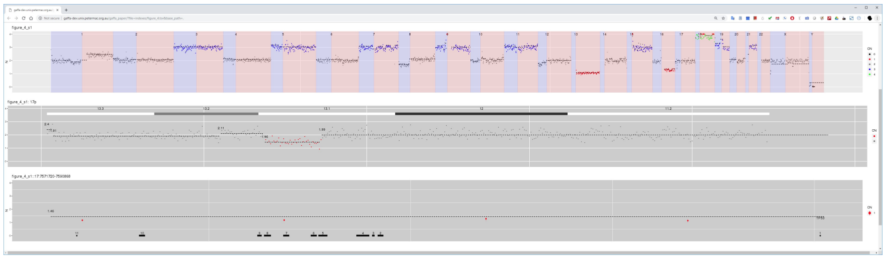

(a)

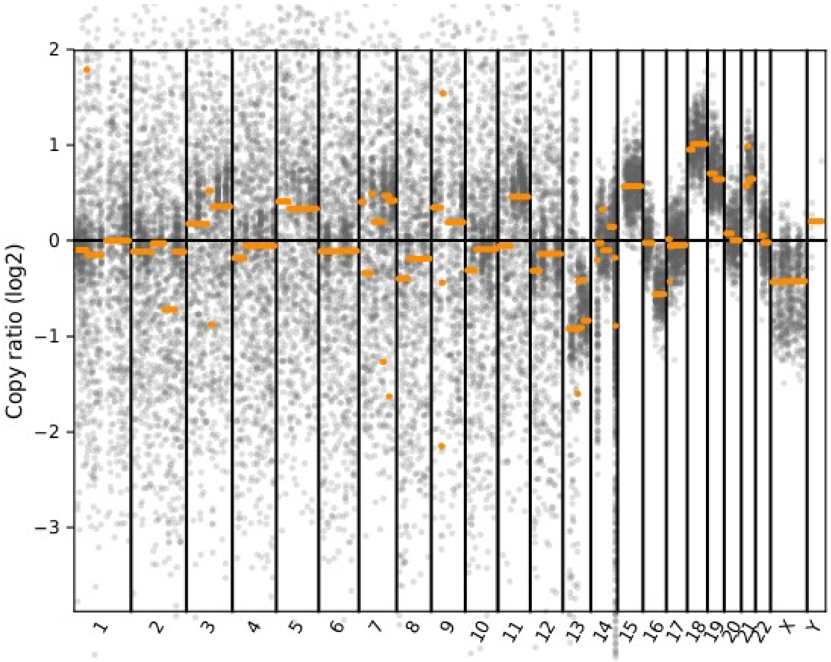


(b)

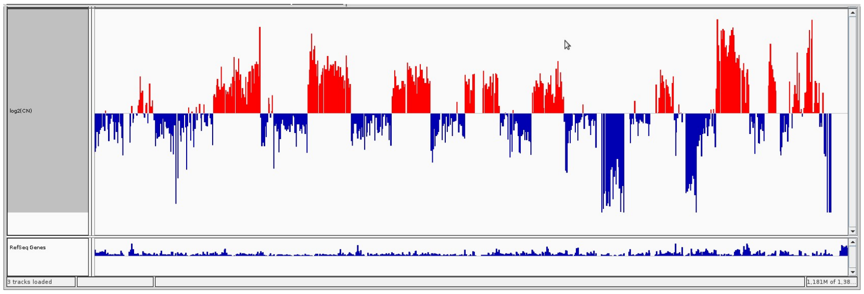

(c)

*Figure 10. Comparison of default data visualisation offered by (a) CNspector, (b) cnvkit and (c) CopywriteR's IgV output track displayed at whole-genome resolution. The final output from canvas is a VCF containing segments at the WG level. While they do display in IgV, they do not render correctly and so we do not include the output.*

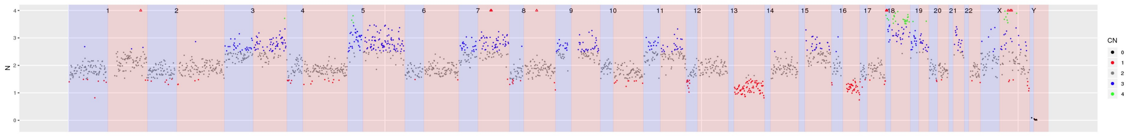

(a)

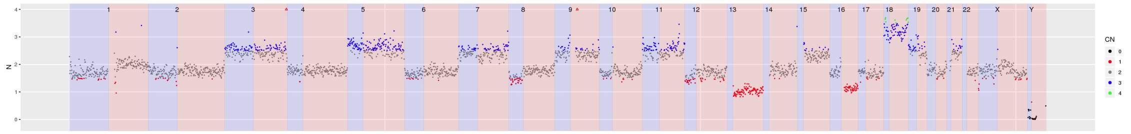

(b)

*Figure 11. (a) CNspector can display outputs derived from canvas, cnvkit and CopywriteR, potentially combining the best features from  each. In this example, CN calls on exons come from canvas, CN calls on WG areas  from CopyeriteR and binned B-allele frequencies and segmented CN after purity correction from CVkit.  (b) Using multi-sample mode enables further improvements to the CN estimates from CopywriteR since the dynamically generated reference contributes less noise to the final CN value than does the single representative sample used by CopywriteR to make the original calls.*

Supplementary Tables

**Table 1**

*DNA library characteristics for patient samples. Reference Samples describe the set of samples originally used to call CN. For multi-sample mode, see main body for description or accompanying web site.*

| Plot | Tissue of origin | Sample type | Tumour/ Germline/ ctDNA | Enrichment | Reads | Reference Samples |
|---|---|---|---|---|---|---|
| Figure 2 | PB | FF | Germline | FRCP | 11243586 | 80 Germline |
| Figure 3 | PB | FF | Germline | FRCP | 11718228 | 80 Germline |
| Figure 4 | BM | FF | Tumour | PHCP | 43533388 | 10 Germline |
| Figure 5 | PB | FF | ctDNA | PHCP | 73792582 | 10 Germline |
| SI Figure 1 | PB | FF | ctDNA | PHCP | 35840940 – 78229626 | 10 Germline |
| SI Figure 2 | PB / BM | FF / FFPE | ctDNA / Tumour | PHCP | 107158934/ 77686656 | 10 Germline |
| SI Figure 3 | NA12878 | Synthetic | Germline | FRCP | 4420342 – 19831926 | 80 Germline |
| SI Figure 4 | PB | FF | Germline | FRCP | 11243586 | 80 Germline |
| SI Figure 5 | BM | FF | Tumour | RNA | 73544995 | 11 Myeloma |
| SI Figure 6 | B | FFPE | Tumour | CCP | 66148862 | 90 Tumour |
| SI Figure 7 | BM | FF | Tumour | None | 104007647 | 3 Tumour |
| SI Figure 8 | B | FFPE | Tumour | CCP | 65809140 | 90 Tumour |
| SI Figure 9 | BM | FFPE | Tumour | AgilentV5 | 146959697 | 8 Tumour |
| SI Figure11 | BM | FF | Tumour | PHCP | | 21 Various |

Abbreviations for tissue of origin: PB=peripheral blood, FF=fresh frozen, BM=bone marrow, FFPE=formalin-fixed paraffin embedded, NA12878=genome in a bottle reference sample, B = biopsy

Abbreviations for enrichment: PHCP=pan-haematology cancer panel, FCRP=familial cancer research panel, CCP=comprehensive cancer panel, RNA=rna-seq, AgilentV5=Agilent Version 5 exome

**Table 2**

Description of targeted sequencing panels used for DNA enrichment of the libraries described in Table 1.

| Name | Genes | Unique bait sequences | Enrichment method | Targets |
|---|---|---|---|---|
| FRCP | 125 | 6071 | Custom dDNA baits on beads | Germline variants in genes conferring cancer risk. |
| PHCP | 363 | 28,764 | Custom DNA baits on beads | Genes and genomic rearrangements in haematological malignancies. |
| CCP | 403 | 54,626 | Custom DNA baits on beads | Pan-cancer somatic variants in actionable genes. |
| AgilentV5 | >50,000 | >500,000 | Commercial kit with DNA baits on beads | All annotated genes. |
| RNA | >20,000 | 1 | Baits on beads | All expressed genes. |

**Table 3**

*Features implemented in CNspector compared to commonly used CNV display tools (including some analysis tools that also do visualisation).*

| Tool | Multi-scale view | Interactive application | Web-based | Use historical results to assess significance | Dynamic display of sample differences | Dynamic reference generation |
|---|---|---|---|---|---|---|
| Gaffa | ✓ | ✓ | ✓ | ✓ | ✓ | ✓ |
| IgV | ✓ | ✓ | ✓ | ✗ | ✗ | ✗ |
| cnvkit | ✓ | ✗ | ✗ | ✗ | ✗ | ✗ |
| CopywriteR | ✓ | ✗ | ✗ | ✗ | ✗ | ✗ |
| Canvas | ✓ | ✗ | ✗ | ✗ | ✗ | ✗ |

Supplementary Information Section S1

## Generation of data displayed by CNspector

CNspector is agnostic to the tools used to generate the tables that it displays. For completeness, we outline the steps used to generate the figures generated by CNspector.

*Alignment and breakpoint generation* - reads were aligned using subread v1.5.0-p3 against the human genome reference build GRCh37.73. Putative breakpoints were also generated by subread and then tabulated for later display.

*Read abundance estimation* - The R package Rsubread::featureCounts() v1.24.0 was used to generate read abundance for all samples using constant non-overlapping fixed-width bins of width 5,000, 50,000, 1,000,000. For enriched samples, read abundance in targeted regions was estimated using bins defined by the genomic regions of the bait sequences. For un-enriched WG samples and for RNA-seq, read abundance in targeted regions was estimated using bins corresponding to the exons used for annotation.

GC correction of read abundance - read abundance values were GC corrected using Loess fitting. All bins intersecting with the ENCODE blacklisted regions (https://www.encodeproject.org/annotations/ENCSR636HFF/) were removed prior to fitting as were bins in the targeted regions corresponding to baits known not to be distributed with weight a function of GC content.

*Copy number estimation* - for each bin, copy number was estimated to be two times (or one times for X and Y bins in men) the ratio of the normalised count in the bin divided by the median of the normalised counts in the corresponding bin in a set of reference samples. For each resolution of bins in each sample, normalisation was performed by scaling so that the median of the non-zero bins was one. The median absolute deviation (MAD) from the median was estimated for each bin across the reference samples and used to compute the

standard deviation (SD). Under the assumption of normality this was taken to be equal to *1.4826 x MAD*.

*B-allele frequencies* - allele frequencies were extracted using R package Rsubread::exactSNP() v1.24.0

*Copy number segmentation* - putative breakpoints were identified from copy number estimates using R package DNAcopy::do_cbc() v1.48.0 to perform circular binary segmentation on the 50,000 base resolution bins. For each segment, the CN was computed using the weighted mean of the bins in each segment.

Supplementary Information Section S2

## Modification of data displayed by CNspector

*Plotting bins* - all copy number estimates are displayed as points located midway along the genomic range of the corresponding bin. At each bin, error bars are computed by setting the coefficient of variation for the sample equal to that of the reference set. Points are colour coded by the nearest integer as a visual indication only.

*Multi-sample mode* - copy number estimation is performed as described in SI Section 2, using the user-selected samples from the displayed batch as the reference set.

*Minimum displayed read support* - by default features are displayed regardless of the number of reads that support them. Adjusting the minimum displayed read depth slider restricts display to those features with read support greater than the number selected. This can be useful to remove noisy entries that may be cluttering the display or confounding interpretation.

*Breakpoint display* - breakpoints are displayed with an O and one end and an X at the other joined by a line. The log of the read support is displayed but scaled so that all breakpoints fit in the region 0<CN<=4. This gives visual separation for clusters of breakpoints near to each other.

*Displaying allele frequencies* - by default all BAFs are displayed regardless of read support or significance. The reason is that for sparsely sampled regions such as unenriched areas in targeted sequencing, there is a shortage of loci that can be used to reliably determine sequence variants or estimate allele frequencies. For visual inspection, even unreliable estimates, taken together, can give a visual indication of zygosity or sample heterogeneity. If required, the option still exists to remove the poorly supported or noisy BAFs by increasing the minimum displayed read support.

# Supplementary Videos

Video 1 shows annotated screen captures demonstrating how to use CNspector to navigate to CNVs shown in Figures 3-5 of the main text.
